# Exploring the impacts of a coffin-lying experience on life and death attitudes of medical and nursing students: preliminary findings

**DOI:** 10.1186/s12909-022-03975-7

**Published:** 2023-01-05

**Authors:** Ruei-Jen Chiou, Po-Fang Tsai, Der-Yan Han

**Affiliations:** 1 Department of Anatomy and Cell Biology, School of Medicine, College of Medicine, Taipei Medical University, Taipei, Taiwan; 2 Graduate Institute of Humanities in Medicine, College of Humanities and Social Sciences, Taipei Medical University, Taipei, Taiwan; 3 Department of Education and Humanities in Medicine, School of Medicine, College of Medicine, Taipei Medical University, Taipei, Taiwan; 4 Section of Liberal Arts, Center for General Education, Taipei Medical University, Taipei, Taiwan

**Keywords:** Coffin-lying, Death attitude, Death education, Medical education

## Abstract

**Background:**

Physicians and nurses often exhibit strong negative emotional and behavioral reactions when patients they care for die, and death education helps them cope with these difficulties. When implementing death education, the literature shows that experiential activities are more effective than lecturing, and progressive exposure is the best way to reduce death anxieties. This study examined the effects of coffin-lying, an activity sometimes seen in Asian cultures, on life and death attitudes of medical and nursing students.

**Methods:**

During a period from 2020 to 2021, 134 medical and nursing students from a medical university in northern Taiwan voluntarily participated in this study. Among them, 53 were in the experimental group, who participated in a coffin-lying activity for nearly 3 hours, and the other 81 were in the control group. All participants filled out questionnaires 1 week before the activity (T1), 1 week after the activity (T2), and 6 ~ 11 weeks after the activity (T3). Three waves of data were analyzed by a repeated-measure multivariate analysis of variance (MANOVA).

**Results:**

The effects of “love and care” and “feeling of existence” were only manifested at T2, however, the scores of “fear of death” and “death avoidance” between the experimental and control groups significantly differed at T2 and T3. In addition, there were no significant differences between the experimental and control groups in “neutral acceptance”, “approach acceptance”, or “escape acceptance”.

**Conclusions:**

The coffin-lying activity based on desensitization was effective in improving “fear of death” and “death avoidance”, and the effects were sustained to 6 ~ 11 weeks. Coffin-lying is not only a well-designed activity that quickly reduces negative tendencies toward death, but it is also worth adopting by medical and nursing schools to make death education more comprehensive.

## Introduction

Dying and death are difficult topics for medical professionals to discuss with patients and patients’ families. Physicians would rather focus on treatments instead of facing the fact that death may occur [1]. One study pointed out that when a physician first encounters the inevitability of the death of a patient under his or her care, he or she may experience different kinds of negative feelings, which, if not handled properly, may cause the physician to respond to the situation by seeming to be unconcerned or numb, keeping their distance, avoiding care, or ignoring the patient’s feelings which could result in an inability to treat the patient properly or avoid medical disputes [2]. As for nurses, their tasks often require frequent, intensive, and in-depth interactions with patients, and therefore, when patients die, they may have strong emotional or behavioral responses, such as fear, loss, shock, emotional crises, avoidance of caring for dying patients, and burnout [3, 4]. Even in practice, nursing students often feel scared and sad during the end-of-life care process and want to share their feelings with other people to calm their emotions and receive support [5, 6].

Death-related courses and activities can be helpful in alleviating these negative reactions. For example, a 3-day introductory oncology course made medical students feel more comfortable by focusing on how to live with cancer and feel less afraid of dealing with death, and thus they were better able to cope with uncomfortable emotional situations [7]. Meta-analyses have shown that end-of-life educational interventions are effective in improving nurses and nursing students’ attitudes toward death [8]. In fact, death education is actually needed for nursing and medical students. A study found that 80.1% of undergraduate nursing students had experienced a patient’s death, and 81.9% of nursing students thought that the number of classes in the field of preparing to deal with patient-death situations was insufficient, so the need to introduce end-of-life care education at a very early stage of education was pointed out [6]. In a 3-year research report, it was mentioned that many physicians cannot properly handle death issues, and it may take them a long time to recover from the loss. Therefore, many senior physicians strongly suggested that it is necessary to implement death education during the student years [7]. Overall, medical and nursing students often encounter patients dying or death during their internship, and it seriously negatively impacts them [5, 6, 8, 9]. Thus, it is essential to strengthen death education for medical and nursing students to deepen their exploration of death, so as to develop self-awareness of death and prevent them from having negative attitudes when they encounter a patient’s death in their future clinical work.

Death education can be carried out through formal theoretical didactic teaching, combined with group discussions, imaginative techniques, role playing, or other experiential activities [10]. However, not all teaching methods achieve the expected outcomes. For example, an early meta-analysis that included 62 studies with randomized controlled designs or non-randomized designs found that death education programs using a didactic approach unexpectedly increased death anxiety when they were compared to control groups [11]. Another meta-analysis and systematic review study adopted stricter inclusion criteria, examined the effects of 15 randomized controlled trials, and found that all of the included studies had good results in reducing death anxiety, and cognitive behavioral treatments centered on graded exposure therapy were most effective [12]. A common graded exposure involves beginning with a terminal illness event that might result in death, progressing to the final death, followed by an imagined funeral [13]. Sometimes the technique of writing one’s own eulogy and a tombstone inscription was also applied [14]. Other experiential activities can be taken as exposure tasks that are suitable for integration into death education including reading obituaries online or in the newspaper, deliberately seeking information on those around the same age who have died, preparing one’s own will, discussing end-of-life preferences, and even visiting cemeteries or funeral homes [13]. To sum up, researchers advocate that experiential learning is more effective than lecturing in implementing death education [6, 15]. If multiple exposure techniques can be delicately composed and carefully experienced by students in a more-vivid way, more-significant effects can be expected.

In fact, some countries in Asia have adopted similar procedures to allow people to participate in “coffin-lying” activities. This kind of activity exposes people to death in a safe way, allowing participants to learn and understand life and death, and is very popular among the public. For example, a company in Tokyo, Japan, holds a “life-ending” death awareness activity in which participants make an advance will, join in their own memorial ceremony, and lie down in a coffin, which unexpectedly attracted many people’s attention [16]. A healing institution in Seoul, South Korea, a funeral etiquette company, has provided “living funerals” for more than 25,000 people since 2012, and improved the quality of life of participants by allowing them to wear grave clothes, lie in coffins, and simulate life after death [17].

In Taiwan, Jen-Teh Junior College of Medicine, Nursing, and Management has set up death-experience classrooms on campus and has held death-experience activities for almost 10 years. Originally, for the sake of strengthening students’ empathy, a psychologist and social worker from the Department of Funeral Science simulated the psychological state of people who are sick and dying, referred to folk beliefs, customs, and rituals, and gradually constructed a coffin-lying procedure. They discussed the procedure and adjusted instructions along with feedback from students and participants. At the beginning of this death-experiencing activity, participants are invited to change into grave clothes. Under the description and guidance of a videotaped story of a girl with bone cancer who eventually dies, they imagine themselves to be a terminally ill patient. Accompanied by soft music, they are asked to consider to whom they would like to thank, apologize, love, and say goodbye, and to write farewell letters and epitaphs. Participants then enter the second death-experience classroom, which contains 10 teaching coffins. Under dim light, comfortable fragrance, and solemn background music, two facilitators invite participants to read part of their own will and epitaph to intensify the feelings of dying. They also adopt some psychodrama techniques, e.g., role-taking and scene-setting to guide participants in completing the death experience steps of entering the coffin, closing the small window on the cover of the coffin, knocking and sealing the coffin with a hammer, and experiencing rebirth after actually spending 10 minutes in the coffin. Participants can fully experience the quietness and loneliness of death, and go through the complete process from illness to death. Most of the time, they feel deeply touched and nourished by the entire process [18, 19]. The coffins are equipped with an emergency button for participants who feel physically or mentally uncomfortable so that they can stop the experience at any time to ensure safety. Once the button is pressed, one of the facilitators will check on the state of the participant, guide him or her to relax and take a break, or even allow them to skip the remaining part of lying in the coffin.

According to terror management theory, having a self-preservation instinct when realizing that death is inevitable results in a basic psychological conflict that produces terror, and this can be managed by a combination of escapism and creating meaning and value [20]. This is consistent with observed results of the coffin-lying activity in that it may have benefits of increasing love and caring for family and friends, enhancing the meaning of life, and changing attitudes toward death [18, 19]. However, one study showed that the coffin-lying process has only immediate effects in reducing negative perspectives and increasing positive perspectives toward death [21]. Therefore, the present study adopted a well-designed coffin-lying process with visual and auditory aids and employed both death attitude- and life attitude-related questionnaires to examine whether the above-mentioned effects were found after the coffin-lying activity, and through a follow-up test, to understand whether the effects could be maintained for a longer time. We hypothesized that the coffin-lying activity would enhance feelings of love and caring, increase the personal meaning of existence, and reduce the fear of death and death avoidance tendencies for participating medical and nursing students.

## Methods

### Study design and participants

Convenience sampling was applied in the present study. In the compulsory courses of General Psychology for year 1 nursing students and Skeletomuscular System for year 2 medical students, we introduced the coffin-lying study to them and explained the purpose of the research under agreement with the lecturers. Although we approached students through their compulsory courses, they were asked to voluntarily join the study without pressure from the course lecturers. Then, we sent an email to these 242 students, inviting them to participate in the study, with a clear explanation that participating or not participating would not influence their grade in the 77course. Among them, 134 students replied, for a response rate of 55.37% and a rejection rate of 44.63%. Participants who completed three-wave data collections received a gift card worth NT$300 (about US$10). Owing to a lack of previous similar studies, we were unable to calculate the sample size in advance. However, if we set a medium effect size (partial η^2^) of 0.06, alpha of 0.05, and power of 0.80 according to general rules, under the condition of two groups and seven response variables, the G*Power program calculated the sample size to be 248, more than the 134 participants in our present study [22, 23]. Because of the insufficient sample size, this research was just a preliminary study that explored the effects of a death-experiencing activity on nursing and medical students.

**Fig. 1:**
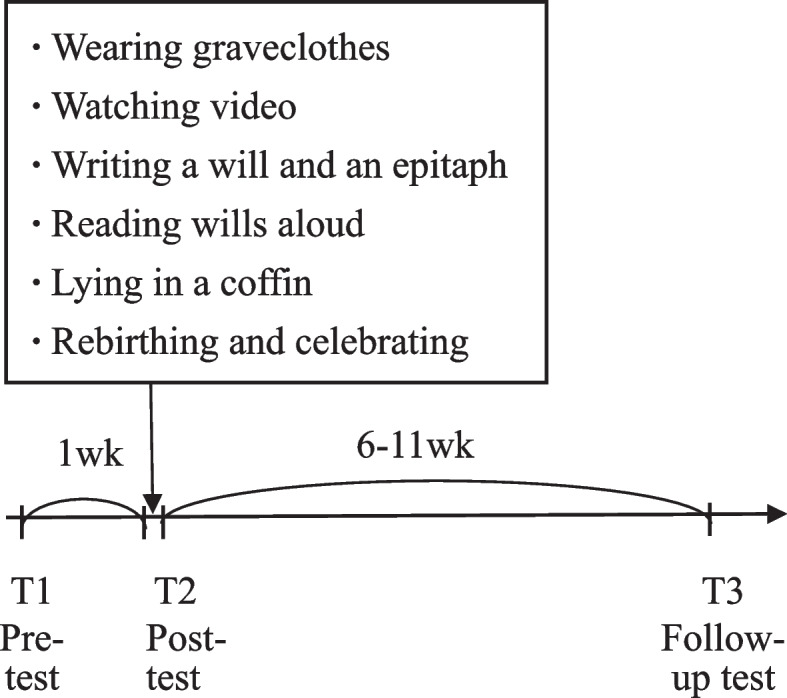
Timeline of the tests and tasks for the experimental group

During the period from October 2020 to June 2021, 65 nursing students and 69 medical students separately participated in the study in two semesters, and they were assigned to an experimental or control group based on their willingness to experience death and their time schedule. Those who were willing or available to lie down in the coffin were assigned to the experimental group, and those who were unwilling or unavailable to lie down in the coffin were assigned to the control group. The group assignment procedure which respected students’ willingness resulted in unequal groups. Participants in both the experimental and control group were administered pre-test questionnaires at the beginning of the semester (T1). After 1 week, students in the experimental group participated in the death-experience activity and received post-test reminder emails after they returned home (T2); control-group participants received reminder emails to complete the questionnaire on the same day as did experimental-group participants. After 6 ~ 11 weeks, both groups completed a follow-up test. The timeline of the experiment is shown in Fig. 1

A psychologist and social worker carried out the coffin-lying exercise for almost 10 years without standardized procedures, and constantly guided participants to complete steps including wearing grave clothes, watching a video, writing a will and an epitaph, reading the will aloud, lying in a coffin, rebirthing, and celebrating. In our experiments, most participants followed the guidance of the facilitators and completed the above-described tasks. However, one student pressed the emergency button shortly after lying in the coffin. A facilitator came to check the situation, and let the student take a break and skip the part of lying in the coffin. That student and the other students had no other unpleasant reactions during our experiments.

### Questionnaires

#### Death attitude profile–revised (DAP-R)

The DAP-R, a multidimensional measure of death-related attitudes among the general public, was developed by Wong et al. in 1994 to assess the extent of (1) fear of death (e.g., “The prospects of my own death arouses anxiety in me.”), (2) death avoidance (e.g., “I avoid death thoughts at all costs.”), (3) neutral death acceptance (e.g., “Death is a natural aspect of life.”), (4) approach acceptance (e.g., “I believe that I will be in heaven after I die.”), and (5) escape acceptance (e.g., “Death will bring an end to all my troubles.”). The questionnaire consists of 32 items and is scored on a 5-point Likert-type scale (1: totally disagree to 5: totally agree). A higher score indicates a stronger attitude toward that dimension. A factor analysis of the scale showed that five factors were identified that explained 66.2% of the total variance, consistent with the original construct and showed good construct validity. The α coefficients of the subscales ranged 0.65 ~ 0.97, indicating an acceptable reliability performance [24]. Studies in Chinese-speaking societies have employed a translated Chinese version of the DAP-R to explore death-related issues, and the text and meaning of the items are suitable for Taiwanese culture, with good overall reliability and validity [25, 26]. To verify the reliability of the scale, the control group data of T1 were employed, and Cronbach’s α of the five subscales were 0.86, 0.84, 0.67, 0.81, and 0.81, respectively. The control group data of T1 and T2 showed that 1-week retest reliabilities for the five subscales were 0.83, 0.75, 0.57, 0.82, and 0.82, respectively.

#### Life attitude inventory (LAI)

The LAI based on the concepts of life formulated by Jean-Paul Sartre, Viktor Frankl, Rollo May, and Carl Rogers was developed by Pan and Hsieh in Taiwan [ 27]. It is utilized to evaluate expectations, attitudes, and actions toward death, perceptions of interactions with others, actions, and the extent to which they could affirm the meaning and value of their existence. The inventory consists of 70 items and is scored on a 7-point Likert-type scale (1: totally disagree to 7: totally agree), with a higher score indicating a more-mature attitude toward life. Cronbach’s α of the overall scale was 0.93, and the reliability of the retest after 4 weeks was 0.91. Exploratory factor analyses and hierarchical clustering analyses confirmed its six-dimensional structure: ideals of life, autonomy, love and care, feeling of existence, attitudes toward death, and life experience. We employed two subscales, “love and care” and “feeling of existence”, to verify the hypothesis in this study. The former consists of 13 items assessing interpersonal attitudes toward other people and behaviors adopted when they interact with people (e.g., “I am willing to spend time to accompany people who need comfort” and “I usually approach others actively and accept them”) with Cronbach’s α of 0.89, and a 1-week retest reliability of 0.84 based on data from the control group in the present study. The latter subscale consists of 11 items assessing the extent to which an individual recognizes the meaning and value of his or her existence (e.g., “I know whom I live for and why I live” and “I often feel it is worth being happy to be alive”), with Cronbach’s α of 0.93, and a 1-week retest reliability of 0.83 in the present study.

### Statistical analysis

Descriptive statistics were applied to present demographic characteristics of participants. Prior to examining the experimental effects, *χ*^*2*^ tests and independent-sample *t*-tests were used to compare differences between the experimental and control groups. Experimental effects were analyzed using a repeated-measure multivariate analysis of variance (MANOVA). First of all, the time effect (within factor), group effect (between factors), and group × time interaction of the seven variables were examined, and the Huynh-Feldt adjustment was used if violations of sphericity were found. We then scrutinized the simple main effects of the time factor (pre/post/follow-up) and group factor (experimental/control) only if the group-time interaction was significant, and analyzed changes during the time or between groups in terms of plotted trends. IBM SPSS vers. 25 was used for all statistical analyses.

### Ethical approval and consent

All methods of the present study were carried out in accordance with relevant guidelines and regulations, and all experimental protocols received ethical approval from Taipei Medical University–Joint Institutional Review Board (N202003108). All students voluntarily participated in the study, and written informed consent was obtained from all participants or their legal guardians.

## Results

All of the participants were assigned to the experimental group or control group based on their willingness to be a part of the coffin-lying activity, so we examined differences in demographic variables (gender, age, and religion) and the independent variable (experimental or control group) through *χ*^*2*^ tests or paired *t*-tests. Similarly, we also scrutinized relationships between six death-relevant variables (death experiences, talking about death in the family, talking about death with friends, religious participation level, believing in life after death level, and participation in funeral rituals) and the independent variable (experimental or control group). Results in Table 1 show that no statistically significant differences existed between the experimental and control groups in terms of demographic variables or death-relevant variables. These findings indicate that students’ decisions to participate or not participate in the coffin-lying activity might not have likely been influenced by their gender, age, or religion (both belonging and participating), and the decision was also independent of the students’ death-related experiences and belief in life after death. Based on these results, we included none of these demographic variables in the MANOVA as covariance factors.

**Table 1:** Demographic variables of the experimental and control groups (*N* = 134)

Variable	Experimental group (*N =* 53)	Control group (*N =* 81)	*χ*^*2*^/*t*	*P*
	*n* (%)	*n* (%)		
Gender			2.435	0.119
Male	14 (26.42%)	32 (39.51%)		
Female	39 (73.58%)	49 (60.49%)		
Age (mean ± SD)	20.94 ± 3.63	21.31 ± 3.39	- 0.593	0.554
Do you have any death experiences of yourself or others?			1.098	0.778
Completely no	9 (16.98%)	10 (12.35%)		
Yes, but no important influence	26 (49.06%)	37 (45.68%)		
Yes, with some influence	12 (22.64%)	23 (28.40%)		
Yes, with important influence	6 (11.32%)	11 (13.58%)		
Did your family talk about death in the past?			3.709	0.295
Never	6 (11.32%)	7 (8.64%)		
Avoided talking unless necessary	21 (39.62%)	28 (34.57%)		
Talked, but not naturally	6 (11.32%)	20 (24.69%)		
Talked in an open atmosphere	20 (37.74%)	26 (32.10%)		
Did you talk about death with your friends in the past?			2.420	0.490
Never	5 (9.43%)	13 (16.05%)		
Avoided talking unless necessary	8 (15.09%)	17 (20.99%)		
Talked, but not naturally	11 (20.75%)	15 (18.52%)		
Talked in a natural atmosphere	29 (54.72%)	36 (44.44%)		
Religion			5.952	0.311
Buddhism	9 (16.98%)	11 (13.58%)		
Folk religion	21 (39.62%)	21 (25.93%)		
I-Kuan Tao	0 (0.00%)	1 (1.23%)		
Christian/Catholic	3 (5.66%)	8 (9.88%)		
None	18 (33.96%)	39 (48.15%)		
Others	2 (3.77%)	1 (1.23%)		
Religious activities participation level (mean ± SD)	2.21 ± 0.86	2.16 ± 0.84	0.313	0.755
Do you believe in life after death? (mean ± SD)	3.40 ± 0.88	3.06 ± 1.07	1.898	0.060
Have you ever participated in funeral rituals?			0.003	1.000
Yes	43 (81.13%)	76 (83.52%)		
No	10 (18.87%)	15 (16.48%)		

SD standard deviation

### Differences in the dependent variables among the three time points

Results of the repeated-measure MANOVA showed that the interaction of dependent variable, group, and time reached a significant level (Table 2), and it was then bifurcated among our seven dependent variables. First of all, in Table 3 there were no significant group × time interaction effects for “neutral acceptance”, “approach acceptance”, or “escape acceptance”, but significant group × time interactive effects with non-significant effects of group or time in both “love and care” (*F*_(1.92, 253.423)_ = 3.256, *p* < 0.05) and “feeling of existence” (*F*_(2, 264)_ = 6.718, *p* < 0.05). Given that we looked at the interaction effect of between-factor (group) and within-factor (time) rather than a single factor in the repeated-measure ANOVA, we ignored the significant elevated effects of time in “approach acceptance” (*F* = 6.606, *p* < 0.01) and “escape acceptance” (*F* = 3.697, *p* < 0.05).

**Table 2:** Results of the multivariate analysis of variance of the seven dependent variables

Effect	SS	Value	F	Hypothesis df	Error df	*P*	Partial eta squared
DV × Group × Time	Pillai’s Trace	0.194	2.433	12.000	121.000	0.007	0.194
	Wilks’ Lambda (λ)	0.806	2.433	12.000	121.000	0.007	0.194
	Hotelling’s Trace	0.241	2.433	12.000	121.000	0.007	0.194
	Roy’s Largest Root	0.241	2.433	12.000	121.000	0.007	0.194

DV dependent variable, SS sum of squares, DF degree of freedom

**Table 3:** Two-way mixed repeated-measure analysis of variance of the dependent variables

Source of variation	SS	df	MS	*F*	*P*	Partial eta squared
**Love and care**						
Group	0.925	1	0.925	1.533	0.218	0.011
Time	0.599	1.920	0.312	2.139	0.122	0.016
Group×Time	0.912	1.920	0.475	3.256*	0.042	0.024
Block	79.610	132	0.603			
Residual	36.968	253.423	0.146			
Total	239.461	391.181				
**Feeling of existence**						
Group	0.002	1	0.002	0.002	0.968	0.000
Time	0.411	2	0.205	0.983	0.375	0.007
Group×Time	2.806	2	1.403	6.718**	0.001	0.048
Block	131.845	132	0.999			
Residual	55.144	264	0.209			
Total	119.208	401				
**Fear of death**						
Group	1.605	1	1.605	3.153	0.078	0.023
Time	3.216	1.768	1.819	12.432***	0.000	0.086
Group×Time	1.276	1.768	0.722	4.933*	0.011	0.036
Block	67.170	132	0.509			
Residual	34.148	233.412	0.146			
Total	107.415	369.948				
**Death avoidance**						
Group	3.161	1	3.161	5.995*	0.016	0.043
Time	2.501	2	1.251	7.625**	0.001	0.055
Group×Time	1.590	2	0.795	4.847**	0.009	0.035
Block	69.600	132	0.527			
Residual	43.300	264	0.164			
Total	120.152	401				
**Neutral acceptance**						
Group	0.052	1	0.052	0.291	0.591	0.002
Time	0.069	2	0.034	0.279	0.757	0.002
Group×Time	0.153	2	0.077	0.624	0.537	0.005
Block	23.667	132	0.179			
Residual	32.432	264	0.123			
Total	563.70	401				
**Approach acceptance**						
Group	0.207	1	0.207	0.482	0.489	0.004
Time	1.300	1.912	0.680	6.606**	0.002	0.048
Group×Time	0.587	1.912	0.307	2.984	0.055	0.022
Block	56.570	132	0.429			
Residual	25.985	252.366	0.103			
Total	84.649	389.19				
**Escape acceptance**						
Group	0.204	1	0.204	0.360	0.550	0.003
Time	1.292	2	0.646	3.697*	0.026	0.027
Group×Time	0.012	2	0.006	0.035	0.966	0.000
Block	74.890	132	0.567			
Residual	46.119	264	0.018			
Total	122.517	401				

* *p* < 0.05 ** *p* < 0.01 *** *p* < 0.001, SS sum of squares, DF degree of freedom, MS mean sum of squares

When checking the simple main effect in advance, we found a slight difference between the two variables. Based on the interaction effect between group and time being significant, a simple main effect of group factor in Table 4 was examined (*F*_(1, 396)_ = 4.250, *p* < 0.05), which indicated a significant difference in the post-test between the experimental (5.400 ± 0.801) and control groups (5.096 ± 0.855) in “love and care”. On the other hand, we found a simple main effect of the time factor in Table 4 in “feeling of existence” (*F*_(2, 264)_ = 5.134, *p* < 0.01), which indicated significant differences between the pre-test (4.761 ± 1.134) and post-test (5.010 ± 1.083), and also between the post-test (5.010 ± 1.083) and follow-up test (4.766 ± 1.199). These findings in Table 4 indicate that the two positive attitudes, “love and care” and “feeling of existence” from the Life Attitude Inventory had their own interaction effects between group and time, while only “feeling of existence” had a simple main effect of a time factor.

**Table 4:** Simple main effects of coffin-lying on the dependent variables

Source of variation	SS	df	MS	*F*	*P* ^a^
**Love and care**					
Group (A factor)					
Pre-test	0.182	1	0.182	0.261	0.610
Post-test	2.960	1	2.960	4.250*	0.040
Follow-up test	0.545	1	0.545	0.783	0.377
Residual	275.796	396	.696		
Time (B factor)					
Experiment group	0.814	1.973	0.413	2.804	0.095
Control group	0.665	1.853	0.359	2.437	0.120
Residual	36.967	250.834	.147		
**Feeling of existence**					
Group (A factor)					
Pre-test	0.627	1	0.627	0.556	0.456
Post-test	1.950	1	1.950	1.727	0.190
Follow-up test	0.234	1	0.234	0.2074	0.649
Residual	450.678	396	1.128		
Time (B factor)					
Experiment group	2.144	2	1.072	5.134**	0.006
Control group	0.790	2	0.395	1.891	0.153
Residual	55.144	264	0.209		
**Fear of death**					
Group (A factor)					
Pre-test	0.119	1	0.119	0.1999	0.655
Post-test	2.867	1	2.867	4.818*	0.029
Follow-up test	3.104	1	3.104	5.216*	0.023
Residual	235.66	396	0.595		
Time (B factor)					
Experiment group	3.522	1.636	2.153	14.567***	0.000
Control group	0.296	1.824	0.162	1.096	0.296
Residual	34.147	230.963	0.148		
**Death avoidance**					
Group (A factor)					
Pre-test	0.670	1	0.670	1.052	0.306
Post-test	6.664	1	6.664	10.468**	0.001
Follow-up test	3.739	1	3.739	5.873*	0.016
Residual	252.099	396	0.637		
Time (B factor)					
Experiment group	3.265	2	1.632	9.951***	0.000
Control group	0.182	2	0.091	0.555	0.575
Residual	43.3	264	0.164		

* *p* < 0.05 ** *p* < 0.01 *** *p* < 0.001, SS, sum of squares, DF degree of freedo, MS mean sum of squares

^a^ Adjustment for multiple comparisons: Bonferroni

Second, more-important results of the two-way mixed ANOVA were significant findings in the “fear of death” and “death avoidance” variables. However, each of them had different situations in both the ANOVA and the simple main effect analysis. We respectively present their statistical information in Tables 2, 3, and 4 with line-graphs of the estimated marginal means of measure.

### Fear of death

**Fig. 2:**
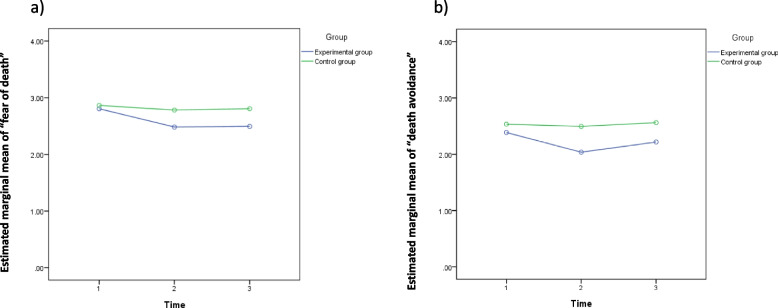
The trends of estimated marginal means of **a**) “fear of death” and **b**) “death avoidance”

For the variable “fear of death” in Table 3, the group × time interaction effect was significant (*F*_(1.768, 233.412)_ = 4.933, *p* < 0.05), even as the time effect was also significant (*F*_(1.768, 233.412)_ = 12.432, *p* < 0.001), so that the simple main effect was examined. The simple main effect of group in Table 4 showed that the means of “fear of death” between the experimental and control groups reached significant differences in the post-test (*F*_(1, 396)_ = 4.818, *p* < 0.05) and follow-up test (*F*_(1, 396)_ = 5.216, *p* < 0.05). Mean comparisons showed that the post-test mean for the experimental group (2.482 ± 0.799) was significantly lower than that for the control group (2.781 ± 0.766, *p* < 0.05), while the follow-up test mean for the experimental group (2.493 ± 0.776) was significantly lower than that for the control group (2.804 ± 0.765, *p* < 0.05). In addition, mean comparisons also showed that within the experimental group (*F*_(1.636, 230.963)_ = 14.567, *p* < 0.001), there were significant differences between the pre-test (2.803 ± 0.789) and post-test (2.482 ± 0.799), and also between the pre-test (2.803 ± 0.789) and follow-up test (2.493 ± 0.776). Trends of the estimated marginal means of “fear of death” by group are shown in Fig. 2a.

### Death avoidance

For the variable “death avoidance” in Table 3, the group × time interaction effect was significant (*F*_(2, 264)_ = 4.847, *p* < 0.05), and the group and time effects were also significant (*F*_(1, 264)_ = 5.995, *p* < 0.05; *F*_(2, 264)_ = 7.625, *p* < 0.01, respectively), so the simple main effect was examined. The simple main effect of group in Table 4 showed that the means of “death avoidance” between the experimental and control groups reached significant differences for the post-test (*F*_(1, 396)_ = 10.468, *p* < 0.01) and follow-up test (*F*_(1, 396)_ = 5.873, *p* < 0.05). Mean comparisons showed that the post-test mean for the experimental group (2.037 ± .729) was significantly lower than that for the control group (2.493 ± .793, *p* < 0.05), while the follow-up test mean for the experimental group (2.218 ± .857) was significantly lower than that for the control group (2.56 ± .865, *p* < 0.05). In addition, mean comparisons also showed that within the experimental group (*F*_(2, 264)_ = 9.951, *p* < 0.001), there was a significant difference between the pre-test (2.388 ± .742) and post-test (2.037 ± .729). The trends of the estimated marginal means of “death avoidance” by group are shown in Fig. 2b.

## Discussion

Although it was observed that the coffin-lying activity had effects of enhancing the extent of “love and care” for family and friends and elevating participants’ “feeling of existence” [18, 19], empirical results of the present study showed that there were only short-term effects after students participated in the coffin-lying activity. The difference in the means of the variable “love and care” between the experimental and control groups at the post-test reached a significant level. In addition, the mean of “feeling of existence” slightly increased at the post-test, but dropped at the follow-up test in the experimental group. This suggests that the coffin-lying activity did not have long-term effects of bringing family or friends closer or enhancing personal life meaning. Through a combination of escapism and creating meaning and value, terror management theory predicts that death-related terror can be managed by a self-preservation instinct [20]. It is possible that terror produced by objects or the atmosphere in the death-experience classroom was soon desensitized in the subsequent coffin-lying process, so that students cherished family and friendships, and enhanced their meaning of life for only a short period of time. However, according to Kolb’s experiential learning theory [28], the best way to learn things is by actually having experiences, and then reflecting and abstracting help by applying the experience to a new environment. We speculated that the effects of enhancing “love and care” and “feeling of existence” might be manifested if this experiential activity were to add some cognitive sparkles or reflective enlightenment. Thus, group discussions or reflective writing are suggested to complement the coffin-lying activity to strengthen the effects of death education in future studies.

Differences in the dependent variables of “fear of death” and “death avoidance” between the experimental and control groups at the post-test and follow-up test were significant, indicating that after the coffin-lying activity, both students’ negative feelings and avoidance tendencies toward death were reduced, and the effects lasted for at least 6 ~ 11 weeks. This finding is consistent with the literature which shows that graded exposure to death is effective in decreasing death anxiety [29]. With peaceful music, a comfortable fragrance, and gentle guidance of the facilitators, the coffin-lying activity allowed students to gradually desensitize their death anxieties. From wearing grave clothes, watching a video, writing a will and epitaph, reading the will and epitaph aloud, lying in a coffin, and celebrating rebirth, students’ fear of death was reduced, and they could face death more openly instead of avoiding it. However, it is not certain which of those tasks drove the effect, and perhaps further qualitative methods may be used to explore the possible benefits from different tasks in the future.

Compared to a previous study that implemented Buddhism death lectures in the first stage and coffin-lying in the second stage in a Buddhist university in Japan [21], our study demonstrated that the coffin-lying activity itself had longer effects on “fear of death” and “death avoidance” in a medical university in northern Taiwan. It seems that didactic lectures and experiential learning lead to different outcomes. Lectures on introducing death concepts from various cultures, understanding the dying process, realizing loss, grief, and bereavement, etc. allow students to acquire death-related knowledge; however, the coffin-lying activity causes students to directly and rapidly decrease their death anxiety. Coffin-lying, a well-designed activity that makes death education more comprehensive, is worth integrating into death education of medical and nursing schools worldwide, instead of being limited to Asian countries. However, if it is difficult to build a special classroom with teaching coffins for students to experience the process of death and dying, virtual reality is another option.

Our results showed that the variable “neutral acceptance” exhibited no significant difference between the experimental and control groups at the post-test or follow-up test. It is reasonable to find this result because the facilitators did not emphasize this concept when they conducted the coffin-lying activity. However, our results showed a trend that the “approach acceptance” and “escape acceptance” were elevated with time. We speculated that as time approached the end of the semester, students felt more stressful and the tendency of “approach acceptance” was increased with “escape acceptance,” especially for students who had religious beliefs. So, we further examined the Pearson correlation of “approach acceptance” and “escape acceptance” at T3 and found that the coefficient from religious students was high (*r* = 0.394, *p* < 0.001). As for those non-religious students, the coefficient did not reach a significant level (*r* = 0.035, *p* > 0.05). In addition, we held the coffin-lying activity in 2010 ~ 2011, a time in which students were exposed to a lot of death-related coronavirus disease 2019 (COVID-19) news. This might have also increased the possibility of higher “approach acceptance” and “escape acceptance” that changed with time. However, further studies are needed to clarify the changes in these two variables.

As for limitations of the study, it is a pity that the present study was not a randomized controlled trial, and it only included students who were willing to participate in such an activity. Students who were too anxious about death or not allowed by their parents did not become our participants. It is doubtful whether the effects can be generalized to students who are reluctant or fearful to join in such an activity. The non-probabilistic sampling technique and unequal groups may have led to bias. If an equal-group design and a larger sample size can be adopted, the findings would be more valuable. In addition, the Hawthorne effect, a tendency in some individuals to alter their behavior in response to their awareness of being observed, cannot be ruled out in this study. Despite these weaknesses, the present study is still of value to provide a new way to experience death that decreases death anxiety.

## Conclusions

The purpose of this study was to explore the impacts of the coffin-lying activity on medical and nursing students’ attitudes toward life and death, and results showed that the coffin-lying activity reduced their fear of death and avoidance behaviors not only for a week after the activity, but the effects were also prolonged for 6 ~ 11 weeks. However, the effects of increasing caring for others and life meaning only existed for a short period of time.

The literature shows that when medical caregivers better understand or are more familiar with death, their attitudes toward death are more mature [7, 8], and their attitudes toward assisting patients or families in dealing with death will be better [29, 30]. We speculated that they might be better able to handle their own emotions of loss when facing the actual death of a patient. However, this remains to be examined by understanding long-term benefits of the coffin-lying activity.

Overall, this study proves that coffin-lying was effective in decreasing the fear of death and death avoidance of medical and nursing students. Thus, it is suggested that medical and nursing schools adopt similar procedures to gradually ameliorate the threat of death, especially in some Asian countries in which discussing death is usually taboo. Medical education nowadays is full of excessive technical and non-humanistic training which favors healing over caring [31], and this study sheds light on the future of medical humanities education.

## Data Availability

The datasets generated and analyzed during the current study are not publicly available due to protection of the privacy of subjects and the problem of Chinese character version, but are available from the corresponding author upon reasonable request.
